# Association Between Early Intravenous Fluids Provided by Paramedics and Subsequent In-Hospital Mortality Among Patients With Sepsis

**DOI:** 10.1001/jamanetworkopen.2018.5845

**Published:** 2018-12-14

**Authors:** Daniel J. Lane, Hannah Wunsch, Refik Saskin, Sheldon Cheskes, Steve Lin, Laurie J. Morrison, Damon C. Scales

**Affiliations:** 1 Institute of Health Policy, Management and Evaluation, Dalla Lana School of Public Health, University of Toronto, Toronto, Ontario, Canada; 2 Rescu, Li Ka Shing Knowledge Institute, St Michael’s Hospital, Toronto, Ontario, Canada; 3 Department of Anesthesia, University of Toronto, Toronto, Ontario, Canada; 4 Department of Critical Care Medicine, Sunnybrook Health Sciences Centre, Toronto, Ontario, Canada; 5 Interdepartmental Division of Critical Care, University of Toronto, Toronto, Ontario, Canada; 6 Division of Emergency Medicine, Department of Family and Community Medicine, University of Toronto, Toronto, Ontario, Canada; 7 Sunnybrook Centre for Prehospital Medicine, Sunnybrook Health Sciences Centre, Toronto, Ontario, Canada; 8 Division of Emergency Medicine, Department of Medicine, University of Toronto, Toronto, Ontario, Canada

## Abstract

**Importance:**

Early administration of intravenous fluids is recommended for all patients with sepsis, but the association of this treatment with mortality may depend on the patient’s initial blood pressure.

**Objective:**

To test the association between early administration of intravenous fluids by paramedics and in-hospital mortality among patients with sepsis, accounting for patients’ initial blood pressure.

**Design, Setting, and Participants:**

Cohort study in which multiple analyses were conducted using a 1-year (from April 1, 2015, to March 31, 2016) cohort of 1871 patients with sepsis who were transported to the hospital by paramedics from a large emergency medical services system in Alberta, Canada. Multivariable logistic regression and a propensity-matched analysis adjusting for baseline patient characteristics were used to minimize confounding by indication and test the association between early administration of intravenous fluids by paramedics and in-hospital mortality. Nonparametric additive regression was used to assess the association of early administration of intravenous fluids with prehospital and in-hospital treatment times.

**Exposures:**

Intravenous fluids administered by paramedics at the point of first contact and during transportation to the hospital.

**Main Outcomes and Measures:**

The primary outcome was in-hospital mortality. Secondary outcomes included prehospital and emergency department treatment times.

**Results:**

A total of 1871 patients with sepsis were identified (955 women and 916 men; median age, 77 years [interquartile range, 64-85 years]), with an overall in-hospital mortality of 28.2% (n = 528). More than half of patients (1015 [54.2%]) received intravenous fluids from paramedics; the median volume provided was 400 mL (interquartile range, 250-500 mL). The association of intravenous fluids with mortality depended on the patient’s initial systolic blood pressure (range, 42-222 mm Hg; *P* < .001 for interaction). For example, in a typical patient with an initial systolic blood pressure of 100 mm Hg, intravenous fluids were associated with decreased mortality (odds ratio, 0.73; 95% CI, 0.56-0.95), but for a typical patient with the median initial systolic blood pressure of 125 mm Hg, intravenous fluids were not associated with in-hospital mortality (odds ratio, 1.41; 95% CI, 0.81-2.44). Similar results were obtained in the propensity-matched analysis. The administration of intravenous fluids was associated with increased prehospital time compared with patients who did not receive intravenous fluids (median difference, 3.2 minutes; 95% CI, 1.7-4.7 minutes) but was not associated with time to assessment in the emergency department (median difference, 2.4 minutes; 95% CI, –2.4 to 7.3 minutes).

**Conclusions and Relevance:**

Intravenous fluids provided by paramedics were associated with reduced in-hospital mortality for patients with sepsis and hypotension but not for those with a higher initial systolic blood pressure.

## Key Points

**Question:**

Is early administration of intravenous fluids provided by paramedics associated with reduced in-hospital mortality in patients with sepsis?

**Findings:**

This cohort study of 1871 patients in Canada found an association between early administration of intravenous fluids provided by paramedics and in-hospital mortality that depends on a patient’s initial blood pressure. Administration of intravenous fluids to patients presenting with a low initial systolic blood pressure was associated with reduced odds of mortality but not among patients with a higher initial systolic blood pressure.

**Meaning:**

Clinicians may consider early intravenous fluid treatment only for patients with sepsis and low initial blood pressure.

## Teaser

This cohort study tests the association between early administration of intravenous fluids by paramedics and in-hospital mortality among patients with sepsis, accounting for patients’ initial blood pressure.

## Introduction

Earlier identification of sepsis and initiation of treatment are essential to reducing mortality in patients with sepsis.^1,2,3^ Early intravenous fluid resuscitation is recommended for the management of sepsis,^4^ but the optimal strategy (ie, amount of fluids administered) for providing intravenous fluids remains controversial. Several trials of early, goal-directed therapy bundles supporting these recommendations found a benefit or no harm from intravenous fluid resuscitation,^5,6,7^ yet numerous meta-analyses and observational studies have found conflicting results.^8,9,10,11,12^ Studying the association of intravenous fluid resuscitation with in-hospital mortality independent of other hospital-based resuscitation and treatment efforts, while specifically examining the association of the patient’s initial blood pressure, may help improve our understanding of the potential benefits or harms of the use of intravenous fluids during the initial phase of the management of sepsis.

Paramedics have a unique opportunity to identify patients with sepsis earlier and provide treatment at the point of first contact.^13^ Previous observational studies have found that initiating intravenous fluid treatment during transportation may reduce the time to achieving resuscitation goals^14,15^ and improve mortality for patients with sepsis.^16^ However, a patient’s initial blood pressure is a strong indication for providing intravenous fluids, and initial blood pressure may also influence whether intravenous fluids have a beneficial or harmful outcome. Using a large cohort of patients transported to the hospital by paramedics, we sought to determine whether a patient’s initial blood pressure modifies the association with in-hospital mortality of providing intravenous fluids to patients with sepsis.

## Methods

### Study Design and Setting

We linked a 1-year cohort of all patients transported by a large, provincial emergency medical services (EMS) system between April 1, 2015, and March 31, 2016, with 2 in-hospital administrative databases (the National Ambulatory Care Reporting System [NACRS] and the Canadian Institute for Health Information Discharge Abstract Database) using each patient’s unique health number, birth date, and date of the EMS event. All included patients were transported by an EMS system operated by Alberta Health Services, the primary health authority in the province of Alberta, Canada. This service supports a population of more than 4 million people in an area of 661 848 km^2^, transporting approximately 160 000 patients per year to 84 acute care facilities throughout the province. A 2-tiered ambulance system is used, with basic life support units that have clinicians trained to the level of emergency medical technicians (primary care paramedics) and advanced life support units equipped with at least 1 paramedic trained in advanced care. Both basic life support and advanced life support clinicians are trained in assessing patients’ vital signs and the administration of intravenous fluids (normal saline).^17^ Paramedics in this system are trained to screen patients for sepsis using criteria similar to Systemic Inflammatory Response Syndrome criteria.^18^ This study was reviewed and approved by the University of Calgary Conjoint Health Research Ethics Board and University of Toronto Health Science Research Ethics Boards. Informed consent was waived as all patient data were deidentified. This study followed the Strengthening the Reporting of Observational Studies in Epidemiology (STROBE) reporting guidelines.^19^

### Participants

We identified patients with sepsis using a previously described strategy for Canadian *International Statistical Classification of Diseases and Related Health Problems, Tenth Revision* (*ICD-10CA*) coding^20^ modified to be consistent with the Sepsis-3 (Third International Consensus Definitions for Sepsis and Septic Shock) definition.^21^ Patients were classified as having sepsis if all 3 of the following conditions were present: they received a diagnosis in the emergency department (ED) of infection (identified by a NACRS discharge code of A00-B99), they were admitted to the hospital or died in the ED (ie, excluding patients discharged home or who left without being treated), and they had evidence of organ dysfunction. Organ dysfunction was identified by the presence of altered vital signs consistent with organ dysfunction at the time of presentation to the paramedics (ie, hypoxemia on pulse oximetry, low mean arterial pressure, or altered Glasgow Coma Scale score)^22,23^ or the presence of a sepsis-related organ dysfunction *ICD-10CA* diagnosis code (eg, respiratory, J96.0; cardiovascular, R57.0; renal, N17.0; neurologic, K72.0; and hematologic, D69.5) or relevant organ dysfunction-related *ICD-10CA* procedure code (eg, endotracheal intubation, 1.GZ.31.CAND)^24^ in the ED record.

### Variables

We extracted operational characteristics (eg, time, municipality, dispatch, and transportation priority) and patient characteristics, including physiological measurements (eg, vital signs) and findings on physical examination (eg, patient symptoms and breath sounds on auscultation), from the paramedic electronic medical record. We used the initial measurement for all recorded physiological measures, as these are the measurements most likely to inform subsequent management by paramedics. Suspicion of sepsis by paramedics was identified from the documented chief reported symptom (patient’s perspective) or paramedic impression (paramedic’s perspective) fields in the electronic patient record or if suspected sepsis was selected as the rationale for administering intravenous fluids. We grouped initial patient location by the population density and proximity to health services of the closest municipality to the scene where the ambulance responded (eg, metropolitan, urban, or rural). Transportation distances were estimated for descriptive purposes using the median distance between the municipality and the hospital to which the patient was transported, as has been done previously.^25^

The primary exposure was the provision of intravenous fluids by paramedics, determined by the documentation of the insertion of an intravenous catheter and/or the administration of any volume of crystalloid fluid (referred to as *intravenous any*). The documented rate of fluid administration (referred to as *intravenous rate*) was also extracted from the paramedic electronic medical record and included administration of no intravenous fluid, saline lock only (ie, patients receiving intravenous medications but no additional intravenous fluids), slow infusion to keep vein open (TKVO) only, or intravenous bolus of fluids. The decision to initiate intravenous fluids and the rate of administration was at the discretion of paramedics but guided by provincial medical directives.^18^

Several important confounders of the association between intravenous fluids and mortality were considered. The use of emergency transportation from the scene to hospital was extracted from documentation. The total prehospital time interval was assessed first as an outcome that may be affected by administration of intravenous fluids and then as a potential confounder in our primary analysis of the association of intravenous fluids and mortality. Prehospital time was calculated as the difference between time the ambulance arrived at the scene and time the ambulance arrived at the hospital, both determined automatically by the computerized automated dispatch system.

### Outcomes

The primary outcome was in-hospital mortality, documented in either the NACRS or Canadian Institute for Health Information Discharge Abstract Database. Secondary outcomes included the total volume of intravenous fluids provided by paramedics (if intravenous fluids were initiated), total prehospital time interval, or the time to assessment by a physician after arrival at the hospital. The total volume of intravenous fluids was determined from the documented “total volume” field or by summing all documented bolus volumes. Time to physician assessment was calculated from the difference between initial assessment time by an ED physician and the documented triage time, both from the NACRS.

### Missing Data

Rates of missing data varied, ranging from 0% for patient age to 36% for patient weight. Most vital sign measures (eg, initial blood pressure, respiratory rate, and heart rate) were missing in less than 3% of patients. We performed multiple imputation for all missing variables used in our regression equations using predictive mean matching and bootstrap resampling (n = 200) with replacement.^26^ Rates of missing values for each variable are described in Table 1.^27^ Characteristics of patients missing the primary outcome are described in eTable 1 in the Supplement.

**Table 1.** Characteristics of Patients With Sepsis Stratified by Rate of Intravenous Infusion^a^

Variable	No Intravenous Fluids (n = 714)	Saline Lock (n = 142)	TKVO (n = 382)	Bolus (n = 633)	*P* Value^b^	SMD^b^
**Operational Characteristics**						
Dispatch priority^c^						
Low	165 (23.1)	26 (18.3)	61 (16.0)	96 (15.2)	.004	0.14
Moderate	272 (38.1)	61 (43.0)	147 (38.5)	249 (39.3)		
High	277 (38.8)	55 (38.7)	174 (45.5)	288 (45.5)		
Patient location						
Metropolitan	574 (80.4)	138 (97.2)	351 (91.9)	563 (88.9)	<.001	0.34
Urban	53 (7.4)	0	9 (2.4)	38 (6.0)		
Rural	87 (12.2)	4 (2.8)	22 (5.8)	32 (5.1)		
Unit type						
Basic life support	86 (12.0)	13 (9.2)	39 (10.2)	62 (9.8)	.50	0.05
Advanced life support	628 (88.0)	129 (90.8)	343 (89.8)	571 (90.2)	.50	0.05
Transportation distance, median (IQR), km	12 (9-17)	14 (12-17)	12 (10-17)	12 (9-16)	.009	0.05
Emergency transportation	114 (16.0)	22 (15.5)	69 (18.1)	138 (21.8)	.04	0.09
Prehospital time, mean (SD), min	42 (16)	45 (18)	45 (17)	45 (15)	.003	0.10
**Initial Patient Characteristics**						
Age, median (IQR), y	78 (65-86)	79 (66-86)	78 (65-86)	73 (61-84)	<.001	0.12
Male sex	324 (45.4)	70 (49.3)	194 (50.8)	328 (51.8)	.10	0.07
Weight, mean (SD), kg	81.1 (29)	78.8 (29)	78.3 (22)	76.5 (28)	.10	0.09
Systolic blood pressure, mm Hg						
Mean (SD)	131 (30)	135 (33)	134 (30)	115 (31)	<.001	0.33
≤110	195 (27.3)	31 (21.8)	85 (22.3)	293 (46.3)	<.001	0.32
Strata						
<80	18 (2.5)	5 (3.5)	11 (2.9)	76 (12.0)	<.001	0.34
80-100	100 (14.0)	16 (11.3)	43 (11.3)	140 (22.1)		
101-120	152 (21.3)	30 (21.1)	72 (18.8)	148 (23.4)		
>120	440 (61.6)	91 (64.1)	253 (66.2)	262 (41.4)		
Diastolic blood pressure, mean (SD), mm Hg	74 (21)	76 (21)	75 (20)	67 (21)	<.001	0.24
MAP, mean (SD), mm Hg	92.4 (21)	95.2 (22)	93.8 (22)	81.7 (22)	<.001	0.32
Respiratory rate, mean (SD), breaths/min	24 (9.6)	24 (8.8)	27 (12)	25 (10)	<.001	0.16
Pulse oximetry, median (IQR), % saturation	92 (83-96)	91 (83-95)	88 (81-94)	89 (83-94)	<.001	0.13
Heart rate, mean (SD), beats/min	98 (26)	102 (22)	99 (25)	104 (28)	<.001	0.15
Temperature, mean (SD), °C	36.9 (1.0)	37.1 (1.3)	37.0 (1.1)	37.2 (1.3)	<.001	0.14
Blood glucose, mean (SD), mg/dL	147.9 (67.9)	140.2 (52.2)	151.9 (72.7)	149.1 (80.9)	.49	0.09
Glasgow Coma Scale score, median (IQR)	15 (12-15)	15 (14-15)	14 (12-15)	14 (11-15)	<.001	0.21
Crackles on auscultation	104 (14.6)	30 (21.1)	68 (17.8)	92 (14.5)	.13	0.10
Decreased air entry on auscultation	142 (19.9)	24 (16.9)	113 (29.6)	175 (27.6)	<.001	0.18
Skin						
Clammy	43 (6.0)	10 (7.0)	36 (9.4)	61 (9.6)	.06	0.08
Pale	133 (18.6)	20 (14.1)	99 (25.9)	177 (28.0)	<.001	0.20
Diaphoretic	38 (5.3)	4 (2.8)	34 (8.9)	49 (7.7)	.03	0.15
Turgor abnormal	73 (10.2)	5 (3.5)	45 (11.8)	103 (16.3)	<.001	0.23
Dyspnea	89 (12.5)	17 (12.0)	60 (15.7)	49 (7.7)	.001	0.13
Patient unresponsive	33 (4.6)	0	9 (2.4)	33 (5.2)	.008	0.19
Paramedic impression of sepsis in patient	34 (4.8)	7 (4.9)	17 (4.5)	104 (16.4)	<.001	0.20
Sepsis medical directive selected	25 (3.5)	5 (3.5)	11 (2.9)	87 (13.7)	<.001	0.20
Prehospital Critical Illness score,^27^ mean (SD)	2.6 (1.4)	2.7 (1.2)	3.1 (1.4)	3.3 (1.5)	<.001	0.28
Total intravenous volume, median (IQR), mL	0 (0-0)	0 (0-0)	150 (50-250)	500 (250-600)	<.001	0.96
**Hospital Characteristics**						
Triage time, mean (SD), min	11 (6.7)	12 (6.2)	12 (7.0)	11 (5.5)	.24	0.10
Time to physician assessment, mean (SD), min	69 (72)	78 (69)	67 (74)	62 (70)	.10	0.12
Mechanical ventilation required	58 (8.1)	9 (6.3)	41 (10.7)	83 (13.1)	.009	0.13
ICU admission	101 (14.1)	12 (8.5)	57 (14.9)	125 (19.7)	.002	0.17
Hospital length of stay, median (IQR)	7 (3-14)	6 (4-13)	6 (4-12)	7 (3-12)	.22	0.06
In-hospital mortality	180 (25.2)	26 (18.3)	112 (29.3)	210 (33.2)	<.001	0.19

Abbreviations: ICU, intensive care unit; IQR, interquartile range; MAP, mean arterial pressure; SMD, standardized mean difference; TKVO, “to keep vein open” rate of infusion.

SI conversion factor: To convert glucose to millimoles per liter, multiply by 0.0555.

^a^ Data are presented as number (percentage) of patients unless otherwise indicated. Proportion of patients missing documentation for each measure, if applicable, is as follows: weight, 36%; systolic blood pressure, 1%; diastolic blood pressure, 1%; respiratory rate, 2%; pulse oximetry, 2%; heart rate, 1%; temperature, 8%; blood glucose, 17%; Glasgow Coma Scale score, 3%; and total intravenous volume, 69%.

^b^ Compare differences between all 4 groups.

^c^ Low priority, codes omega/alpha; moderate priority, codes bravo/Charlie; and high priority, codes delta/echo.

### Statistical Analysis

Descriptive statistics are reported with continuous measures expressed as means with SD or medians with interquartile range and categorical variables expressed as frequencies within the group. Simple tests of association comparing these variables, stratified by intravenous fluid rate, were completed using analysis of variance for normally distributed variables, Kruskal-Wallis rank sum test for nonnormally distributed variables, and χ^2^ test for categorical variables. *P* values and standardized mean differences, a measure of the mean difference of the mean SDs, are reported together, as *P* values are more sensitive to small but clinically unimportant differences in large sample sizes. These *P* values were only provided as descriptive measures, so no interpretation of significance was used. All *P* value tests were 2-sided.

Our primary analysis examined the association of the provision of any intravenous fluids by paramedics with in-hospital mortality in a multivariable regression model while accounting for a patient’s initial systolic blood pressure, using an interaction between systolic blood pressure and intravenous fluids. This analysis controlled for several potential confounders of this association, including paramedic’s suspicion of sepsis, total prehospital time, transportation priority, and the patient’s initial illness state (baseline patient characteristics, initial physiological measures, documented symptoms, and findings on physical examination) but excluding measures with multicollinearity (eg, diastolic blood pressure). Restricted cubic splines with 3 knots were used for continuous measures to account for nonlinearity. The possibility of nonindependence of mortality (ie, clustering) across destination hospitals was addressed using Huber-White robust covariance matrix estimates.^26^ To illustrate the effect modification of initial blood pressure across a range of initial values, we estimated the association between provision of intravenous fluids and in-hospital mortality using the median initial blood pressure of the overall population (125 mm Hg) and blood pressure at a commonly accepted threshold for hypotension (systolic blood pressure <100 mm Hg).

We then conducted several secondary analyses using alternative methods to test the association between administration of intravenous fluids and in-hospital mortality. A propensity-matched analysis was conducted by calculating a propensity score for intravenous fluid treatment using the same baseline patient characteristics. This propensity score was used to match patients who received intravenous fluids with patients who did not receive intravenous fluids 1:1 using the nearest neighbor technique with replacement of controls; a final model including all other exposures was estimated, with controls weighted for the number of matches. An overview of our propensity-matched cohort is presented in eAppendix 1, eTable 2, and eFigures 1 and 2 in the Supplement. Finally, an instrumental variable analysis was conducted using intravenous treatment rates per municipality as the instrument, but no residual confounding was identified; these results are described in eAppendix 2, eTables 3 and 4, and eFigures 3 and 4 in the Supplement. Given the use of initial blood pressure as an effect modifier, the adjusted estimates for in-hospital mortality depend on the patient’s initial blood pressure. For illustrative purposes, we provide estimates for the median systolic blood pressure of the overall population and blood pressure at a potentially clinically relevant threshold for hypotension (100 mm Hg).

The 3 secondary outcome measures (total intravenous fluid volume, prehospital time, and time to physician assessment in the ED) were modeled using nonparametric additive regression with bootstrapping, a regression method that provides estimates on the original scale when variables require transformation owing to nonlinearity.^26^ For the total intravenous fluid volume model, we included all baseline patient characteristics, transportation priority, patient location (eg, metropolitan, urban, and rural), and paramedic-documented suspicion of sepsis. For the prehospital time model, we used the same variables as the total intravenous fluid volume model and then added as exposure variables intravenous fluids and operational factors likely to affect prehospital time, including the month and hour of the call for transportation (divided into 24 hours). For the time to physician assessment model, we used the same variables as the prehospital time model and added prehospital time as an exposure. Separate models were created for the intravenous any fluids and intravenous fluid rate exposures. Median difference and 95% CIs for the exposures of interest were estimated using 200 bootstrap resamples.

### Sensitivity Analyses

We performed several sensitivity analyses to test the robustness of our findings (eFigures 5, 6, and 7 and eTable 5 in the Supplement). First, the rate of infusion (ie, saline lock, TKVO, or bolus) was included in the multivariable model as a 4-level factor instead of the binary intravenous fluid treatment variable to identify any dose-response association. To test our multiple imputation, we repeated all primary analysis using only patients with complete measures for all variables. Finally, we repeated our analysis to replicate the analysis completed by a previous study investigating the association between intravenous fluids administered by paramedics and mortality in patients with sepsis.^16^

For all analyses, we report estimates of associations with 95% CIs, where appropriate, and considered results to be statistically significant when the 95% CIs did not include 1. All statistical analyses were completed in R (R Foundation for Statistical Computing)^28^ using “tableone” package for descriptive statistics; “rms” package for regression modeling, imputation, and contrasting variables; “Hmisc” package for nonparametric additive regression; and the “MatchIt” package for propensity matching.^26,29,30^ R code is reported in eAppendix 3 in the Supplement.

## Results

### Participants

**Figure 1.**
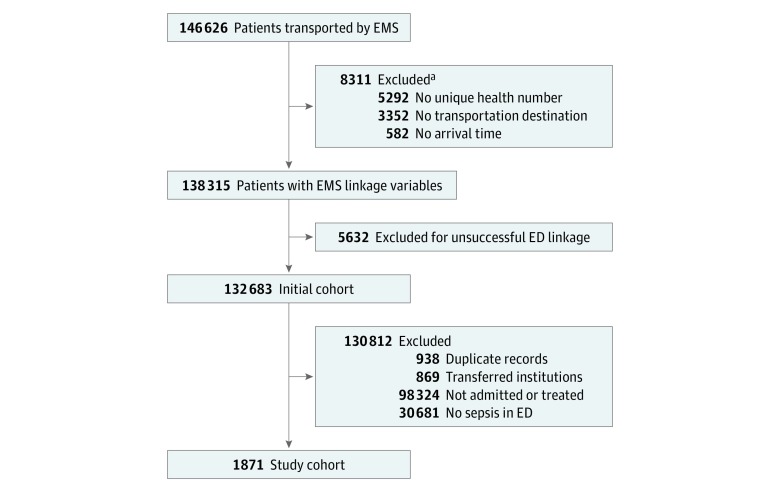
Study Flow Diagram ED indicates emergency department; EMS, emergency medical services. ^a^Not mutually exclusive.

A total of 146 626 adults were transported to the hospital by paramedics during the study timeline, of which 132 683 patients were successfully linked to the NACRS (90.5% linkage rate). Of these patients, 1871 were identified in the ED as having sepsis and composed the primary study cohort (955 women and 916 men; median age, 77 years [interquartile range, 64-85 years]) (Figure 1). More than half of the patients with sepsis received intravenous fluids from paramedics (intravenous any, 1015 [54.2%]), while 142 patients with sepsis (7.6%) received only a saline lock but no intravenous fluids, and 714 patients with sepsis (38.2%) received no intravenous fluids. The overall mortality rate was 28.2% (n = 528); 36 patients died in the ED and 492 patients died in the hospital (Table 1).^27^ Mortality was higher among patients who received any intravenous fluids (322 of 1015 [31.7%]) than among patients who received no intravenous fluids (206 of 856 [24.1%]).

### Process Outcomes

Among patients who received intravenous fluids, the median volume provided was 400 mL (interquartile range, 250-500 mL) (Table 2). Patients for whom paramedics documented a suspicion of sepsis received more fluids than patients with no documented suspicion of sepsis (median difference, 97.0 mL; 95% CI, 1.3-192.0 mL). Patients who received intravenous fluids had longer prehospital times than patients not receiving intravenous fluids (median difference, 3.2 minutes; 95% CI, 1.7-4.7 minutes), with the largest difference observed for patients receiving bolus doses of fluid (median difference, 3.9 minutes; 95% CI, 1.8-6.0 minutes). Once in the ED, the administration of intravenous fluids was not associated with time to assessment (median difference, 2.4 minutes; 95% CI, –2.4 to 7.3 minutes). Patients who received emergency transportation had a reduced time interval to physician assessment compared with those who did not receive emergency transportation (median difference, –29 minutes; 95% CI, –44 to –14 minutes). Patients with low initial systolic blood pressure (<100 mm Hg) also had a reduced time interval to physician assessment compared with those who did not have low initial systolic blood pressure (median difference, –6.4 minutes; 95% CI, –11.0 to –1.4 minutes), but no differences were observed for patients in whom paramedics suspected sepsis or who received intravenous fluids at any rate of administration.

**Table 2.** Median Change in Total Volume of Intravenous Fluids, Total Prehospital Time, and Time to Physician Assessment in the Emergency Department^a^

Covariate	Total Volume, mL (n = 575)	Prehospital Time, min (n = 1871)	Time to Physician Assessment, min (n = 1654)
Crude median change			
Any fluid	400 (250 to 500)^b^	2.5 (1.0 to 3.9)^c^	−6.2 (−13.0 to 0.49)^c^
Saline lock^d^	0 (0 to 0)^b^	2.6 (−0.3 to 5.5)^c^	9.3 (−3.8 to 22.0)^c^
TKVO^d^	150 (50 to 250)^b^	3.0 (1.0 to 5.0)^c^	−1.5 (−11.0 to 7.7)^c^
Bolus^d^	500 (250 to 600)^b^	2.9 (1.1 to 4.6)^c^	−6.5 (−14.0 to 1.4)^c^
Paramedic suspicion of sepsis in patient	500 (300 to 825)^b^	0.9 (−1.7 to 3.5)^c^	−16.0 (−28.0 to −4.0)^c^
Emergency transportation	300 (200 to 500)^b^	−4.9 (−6.8 to −3.1)^c^	−51.0 (−59.0 to −43.0)^c^
Nonparametric additive regression, median change (95% CI)			
Any fluid	NA	3.2 (1.7 to 4.7)	2.4 (−2.4 to 7.3)
Saline lock	NA	2.1 (−1.2 to 5.4)	4.1 (−5.8 to 14.0)
TKVO	NA	3.1 (1.0 to 5.2)	2.2 (−3.5 to 8.0)
Bolus	NA	3.9 (1.8 to 6.0)	3.7 (−3.0 to 10.0)
Paramedic suspicion of sepsis in patient	97 (1 to 192)	1.2 (−1.6 to 4.0)	−0.3 (−9.2 to 8.6)
Emergency transportation	−33 (−86 to 21)	−5.1 (−7.5 to −2.8)	−29.0 (−44.0 to −14.0)
Initial SBP of 100 mm Hg^e^	16 (−17 to 55)	−0.6 (−1.9 to 0.6)	−6.4 (−11.0 to −1.4)

Abbreviations: NA, not applicable; SBP, systolic blood pressure; TKVO, “to keep vein open” rate of infusion.

^a^ Baseline patient characteristics included in each analysis were emergency transportation, unit type, community type (metropolitan, urban, or rural), paramedic suspicion of sepsis in patient, age, sex, weight, SBP, Glasgow Coma Scale score, respiratory rate, heart rate, temperature, blood glucose level, auscultation findings (congested, crackles, wheezing, or decreased air entry), and physical examination findings (skin clammy, pale, diaphoretic, jaundice, turgor, symptoms of malaise, dyspnea, or weakness).

^b^ Median (interquartile range).

^c^ Mean (95% CI).

^d^ Sensitivity analysis using rate of administration of intravenous fluids instead of binary variable of any intravenous fluids.

^e^ Relative to median initial SBP of 125 mm Hg.

### Patient Mortality

**Figure 2.**
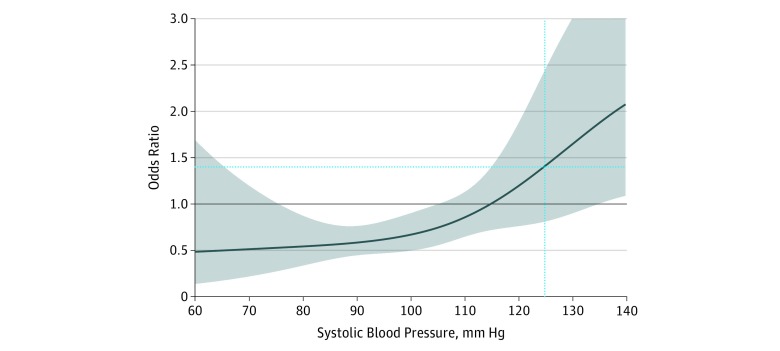
Changes in Odds of Mortality With Intravenous Fluid Treatment at Different Initial Systolic Blood Pressures in a Multivariable Model The gray band indicates the 95% CI. The vertical dotted line indicates the cohort median estimate (ie, adjusted to median systolic blood pressure of 125 mm Hg); the horizontal dotted line indicates the odds ratio for the cohort median estimate.

The association of intravenous fluids with mortality depended on the patient’s initial systolic blood pressure (range, 42-222 mm Hg; *P* < .001 for interaction). In crude analysis, patients who received any fluids had increased odds of mortality (odds ratio [OR], 1.3; 95% CI, 1.0-1.6), as did those who received a bolus dose of fluids (OR, 1.5; 95% CI, 1.2-1.9) (Table 3). In multivariable analysis, the odds of mortality associated with administration of intravenous fluids depended on the patient’s initial systolic blood pressure (analysis of variance test of interaction coefficients *P* < .001). At the median systolic blood pressure for the overall population (125 mm Hg), intravenous fluids were not associated with mortality (OR, 1.4; 95% CI, 0.8-2.4) at any rate of administration. However, an association with decreased mortality was observed for patients who received intravenous fluids when their initial systolic blood pressure was low (eg, <100 mm Hg) (Figure 2). For example, in a typical patient with an initial systolic blood pressure of 100 mm Hg, intravenous fluids were associated with decreased mortality (OR, 0.73; 95% CI, 0.56-0.95), but for a typical patient with the median initial systolic blood pressure of 125 mm Hg, intravenous fluids were not associated with in-hospital mortality (OR, 1.41; 95% CI, 0.81-2.44). This trend was also observed in the sensitivity analysis using rate of administration of intravenous fluids instead of the binary treatment variable of receipt of intravenous fluids, with bolus rates of intravenous fluid administration associated with a greater decrease in mortality than TKVO or saline lock only in patients with low initial systolic blood pressure of 100 mm Hg (bolus: OR, 0.62; 95% CI, 0.45-0.86 vs TKVO: OR, 0.71, 95% CI, 0.32-1.58 vs saline lock: OR, 0.79; 95% CI, 0.41-1.54) (Table 3 and eFigure 1 in the Supplement). Emergency transportation from the scene (OR, 1.17; 95% CI, 0.86-1.60), prehospital time interval (OR, 1.01; 95% CI, 0.90-1.15), and paramedic suspicion of sepsis (OR, 1.15; 95% CI, 0.81-1.63) had no independent association with mortality (eFigure 2 in the Supplement).

**Table 3.** Data on Hospital Mortality Among Patients With Sepsis Treated With Intravenous Fluid by Paramedics Compared With Those With No Intravenous Treatment^a^

Regression Model	Crude OR (95% CI)	Multivariable Adjusted OR (95% CI)^b^	
		Cohort Median	Hypotensive
Any fluid	1.3 (1.0-1.6)	1.40 (0.81-2.44)	0.67 (0.49-0.90)
Saline lock^c^	0.7 (0.4-1.1)	1.35 (0.49-3.69)	0.79 (0.41-1.54)
TKVO^c^	1.2 (0.9-1.6)	1.57 (0.79-3.15)	0.71 (0.32-1.58)
Bolus^c^	1.5 (1.2-1.9)	1.38 (0.68-2.80)	0.62 (0.45-0.86)
Propensity matched	NA	1.41 (0.93-2.14)	0.57 (0.37-0.89)

Abbreviations: NA, not applicable; OR, odds ratio; TKVO, “to keep vein open” rate of infusion.

^a^ Using 40 *df*.

^b^ Estimates of treatment effect adjusted to systolic blood pressure as follows: cohort median, 125 mm Hg; and hypotensive, 100 mm Hg.

^c^ Sensitivity analysis conducted for multivariable model using rate of infusion instead of a binary variable of treatment with intravenous fluids. Variables included in each analysis were the same as those mentioned in Table 2 and prehospital time.

We successfully matched 1489 patients (79.6%; 1015 who received intravenous fluids) for their propensity to receive intravenous fluid treatment based on their baseline characteristics (eFigure 3 in the Supplement). The trend of decreased odds of mortality for lower systolic blood pressure and increased odds of mortality for higher systolic blood pressure was again observed (eFigure 4 in the Supplement), with reduced odds of mortality at an initial systolic blood pressure of 100 mm Hg (OR, 0.57; 95% CI, 0.37-0.89). Overall results were consistent when the analysis was repeated in patients with no missing observations and using a similar approach as a previously published study^16^ (eTable 2 in the Supplement). In the instrumental variable analysis, we found no evidence of residual endogeneity despite a good instrument, supporting the use of only multivariable and propensity-matched techniques to test this association. Results of the instrumental variable analysis are presented in eAppendix 2, eTables 3 and 4, and eFigures 3 and 4 in the Supplement.

## Discussion

### Key Results

In this cohort of EMS-transported patients with sepsis diagnosed in the ED, we found an association between early administration of intravenous fluids and patient mortality that depended on the patient’s initial systolic blood pressure and was independent of the paramedic’s suspicion of sepsis, receipt of emergency transportation from the scene, and total prehospital time interval. Intravenous fluid treatment by paramedics appeared to be beneficial in patients with a low initial systolic blood pressure (eg, <100 mm Hg) but had no association with mortality or may even have been harmful in patients with higher initial systolic blood pressures.

### Interpretation

This study adds to the literature by exploring the association of early administration of intravenous fluids and mortality among patients with sepsis across a range of initial systolic blood pressures. However, defining an explicit threshold for treatment using a continuous measure, such as systolic blood pressure, often does not reflect a biological reality for potential benefit or harm. We therefore present estimates of association at clinically important thresholds to illustrate the change in association but suggest that clinicians should consider these thresholds only as approximate when making treatment decisions. The trend we describe was consistent using multiple analytic approaches and in multiple sensitivity analyses.

Paramedics provided more intravenous fluids for patients in whom they suspected sepsis; although patients who received intravenous fluids had longer prehospital time intervals, these delays were unlikely to be clinically meaningful. Once in the ED, no difference in the time to physician assessment was observed for patients who received intravenous fluids compared with those who did not receive intravenous fluids; however, patients who received emergency transportation or who had low initial systolic blood pressure had reduced time to physician assessment, suggesting that severity of illness was a key indication for expedited treatment in the ED.

Our overall results (ie, adjusted to the median systolic blood pressure of 125 mm Hg) showing no association of early administration of intravenous fluids with mortality differ from results of another large study of EMS-transported patients with severe sepsis that demonstrated a benefit from early administration of intravenous fluids (OR, 0.46; 95% CI, 0.23-0.88).^16^ A key difference in our study was the inclusion of an interaction between initial systolic blood pressure and intravenous fluids in our regression models, which aligns with the hypothesis that patients with hypoperfusion benefit more from early administration of intravenous fluids than do patients with normal perfusion pressures.

### Limitations

Our analysis was limited in several ways. First, a lack of information on additional prehospital interventions performed by paramedics (eg, oxygen therapy or electrocardiographic monitoring) prevented us from including these in our analysis. Previous studies in EMS have reported that nearly all patients with sepsis who receive intravenous fluids also receive these interventions from paramedics,^16^ so they are unlikely to contribute independently to observed associations. Second, we had limited information on in-hospital treatments provided to our patients and were unable to explore the independent association of these treatments with mortality. Definitive treatment (ie, antibiotics) and time to treatment are critical factors contributing to survival in these patients^8^; therefore, we provide an exploration of the association of prehospital factors with time to physician assessment, a surrogate for the earliest time a patient could receive antibiotics, as they are not currently provided by paramedics. Third, we selected patients with sepsis using an algorithm that relies on ED diagnosis codes. These criteria reflect diagnosis in a setting with more diagnostic capabilities than are currently available to paramedics and therefore may not reflect patients with sepsis that is able to be recognized in the prehospital setting. Strategies to improve paramedic recognition of infection and sepsis are required to ensure that the right patients receive the right treatment. Fourth, given the observational design of our study, we are unable to determine whether early administration of intravenous fluids has an independent causal association with mortality in patients with sepsis. The association we noted may be a surrogate for the true causal factor, the result of residual confounding in our analysis, or both. For example, it is possible that the association we note is because patients with a low blood pressure who are administered intravenous fluids are recognized as more critical at ED triage and therefore have a shorter time to receipt of antibiotics in the ED. Planned clinical trials of liberal vs restrictive intravenous fluid treatment for patients with sepsis should help determine the independent association with sepsis mortality.^31,32,33^

## Conclusions

Early administration of intravenous fluids by paramedics was associated with a reduction in mortality among patients with sepsis who had a low initial systolic blood pressure (eg, <100 mm Hg) but not among patients with higher initial systolic blood pressures. Among patients with suspected sepsis, clinicians may consider administering intravenous fluids when the initial systolic blood pressure is low and a more restrictive approach to fluid resuscitation when systolic blood pressure is high.

## Notes

Supplement. **eTable 1.** Descriptive Statistics Comparing Patients Included in Study With Those Excluded Due to Missing Primary Outcome (ie, Patients Transferred From Index Hospital Prior to Discharge) **eTable 2.** Descriptive Characteristics of Propensity-Matched Patient Cohort (n = 1489) **eTable 3.** Characteristics of Patients Across Instrumental Variable Tertiles **eTable 4.** Odds Ratios (95% CI) for Hospital Mortality in Patients With Sepsis Treated With IV Fluid Treatment by Paramedics **eTable 5.** Sensitivity Analysis Odds Ratios (95% CI) for Hospital Mortality in Patients With Sepsis Treated With IV Fluid Treatment by Paramedics **eAppendix 1.** Propensity-Matched Analysis **eAppendix 2.** Instrumental Variable Analysis **eAppendix 3.** R Code **eFigure 1.** Standardized Mean Difference of Baseline Patient Characteristics Used to Generate Propensity Score **eFigure 2.** Changes in Odds of Mortality for IV Fluid Treatment at Different Initial Systolic Blood Pressures, With 95% Confidence Bands, in Propensity Matched Model **eFigure 3.** Proportion of Patients Who Received IV Fluid Across Levels of Instrumental Variable **eFigure 4.** Changes in Odds of Mortality for IV Fluid Treatment at Different Initial Systolic Blood Pressures, With 95% Confidence Bands, in Instrumental Variable Model **eFigure 5.** Changes in Odds of Mortality for IV Fluid Treatment at Different Initial Systolic Blood Pressures, With 95% Confidence Bands, in Sensitivity Analysis Comparing Patients Who Received Fluid at a Bolus Rate With Patients Who Received No IV Fluid **eFigure 6.** Changes in Odds of Mortality for IV Fluid Given Patients Initial Systolic Blood Pressure, Excluding Patients With Missing Measures **eFigure 7.** Odds of In-Hospital Mortality for Additional Measures From Multivariable Model **eReferences**
