# Errors in Odds Ratios

**DOI:** 10.1001/jamanetworkopen.2018.7976

**Published:** 2019-01-25

In the Original Investigation titled “Association Between Early Intravenous Fluids Provided by Paramedics and Subsequent In-Hospital Mortality Among Patients With Sepsis,”^1^ published December 14, 2018, there were errors in 2 odds ratios and a 95% CI in the Results section of the abstract and text. The odds ratio (95% CI) for a typical patient with an initial systolic blood pressure of 100 mm Hg should be changed from 0.73 (95% CI, 0.56-0.95) to 0.67 (95% CI, 0.49-0.90), and the odds ratio for a typical patient with a median initial systolic blood pressure of 125 mm Hg should be changed from 1.41 to 1.40. This article was corrected online.^1^
