# Supplementary material for: Association Between Early Intravenous Fluids Provided by Paramedics and Subsequent In-Hospital Mortality Among Patients With Sepsis
Source: JAMA Netw Open. 2018 Dec 14;1(8):e185845. doi: 10.1001/jamanetworkopen.2018.5845 (PMC6324346; doi:10.1001/jamanetworkopen.2018.5845)
Supplement: Supplement. — eTable 1. Descriptive Statistics Comparing Patients Included in Study With Those Excluded Due to Missing Primary Outcome (ie, Patients Transferred From Index Hospital Prior to Discharge) eTable 2. Descriptive Characteristics of Propensity-Matched Patient Cohort (n = 1489) eTable 3. Characteristics of Patients Across Instrumental Variable Tertiles eTable 4. Odds Ratios (95% CI) for Hospital Mortality in Patients With Sepsis Treated With IV Fluid Treatment by Paramedics eTable 5. Sensitivity Analysis Odds Ratios (95% CI) for Hospital Mortality in Patients With Sepsis Treated With IV Fluid Treatment by Paramedics eAppendix 1. Propensity-Matched Analysis eAppendix 2. Instrumental Variable Analysis eAppendix 3. R Code eFigure 1. Standardized Mean Difference of Baseline Patient Characteristics Used to Generate Propensity Score eFigure 2. Changes in Odds of Mortality for IV Fluid Treatment at Different Initial Systolic Blood Pressures, With 95% Confidence Bands, in Propensity Matched Model eFigure 3. Proportion of Patients Who Received IV Fluid Across Levels of Instrumental Variable eFigure 4. Changes in Odds of Mortality for IV Fluid Treatment at Different Initial Systolic Blood Pressures, With 95% Confidence Bands, in Instrumental Variable Model eFigure 5. Changes in Odds of Mortality for IV Fluid Treatment at Different Initial Systolic Blood Pressures, With 95% Confidence Bands, in Sensitivity Analysis Comparing Patients Who Received Fluid at a Bolus Rate With Patients Who Received No IV Fluid eFigure 6. Changes in Odds of Mortality for IV Fluid Given Patients Initial Systolic Blood Pressure, Excluding Patients With Missing Measures eFigure 7. Odds of In-Hospital Mortality for Additional Measures From Multivariable Model eReferences [file jamanetwopen-1-e185845-s001.pdf]

Supplementary Online Content

Lane DJ, Wunsch H, Saskin R, et al. Association between early intravenous fluids provided by paramedics and subsequent in-hospital mortality among patients with sepsis. *JAMA Netw Open*. 2018;1(8):e185845.
doi:10.1001/jamanetworkopen.2018.5845

eTable 1. Descriptive Statistics Comparing Patients Included in Study With Those Excluded Due to Missing Primary Outcome (ie, Patients Transferred From Index Hospital Prior to Discharge)
eTable 2. Descriptive Characteristics of Propensity-Matched Patient Cohort (n=1489)
eTable 3. Characteristics of Patients Across Instrumental Variable Tertiles
eTable 4. Odds Ratios (95% CI) for Hospital Mortality in Patients With Sepsis Treated With IV Fluid Treatment by Paramedics
eTable 5. Sensitivity Analysis Odds Ratios (95% CI) for Hospital Mortality in Patients With Sepsis Treated With IV Fluid Treatment by Paramedics
eAppendix 1. Propensity-Matched Analysis
eAppendix 2. Instrumental Variable Analysis
eAppendix 3. R Code
eFigure 1. Standardized Mean Difference of Baseline Patient Characteristics Used to Generate Propensity Score
eFigure 2. Changes in Odds of Mortality for IV Fluid Treatment at Different Initial Systolic Blood Pressures, With 95% Confidence Bands, in Propensity Matched Model
eFigure 3. Proportion of Patients Who Received IV Fluid Across Levels of Instrumental Variable
eFigure 4. Changes in Odds of Mortality for IV Fluid Treatment at Different Initial Systolic Blood Pressures, With 95% Confidence Bands, in Instrumental Variable Model
eFigure 5. Changes in Odds of Mortality for IV Fluid Treatment at Different Initial Systolic Blood Pressures, With 95% Confidence Bands, in Sensitivity Analysis

© 2018 Lane DJ et al. *JAMA Network Open.*

Comparing Patients Who Received Fluid at a Bolus Rate With Patients Who Received No IV Fluid
eFigure 6. Changes in Odds of Mortality for IV Fluid Given Patients Initial Systolic Blood Pressure, Excluding Patients With Missing Measures
eFigure7. Odds of In-Hospital Mortality for Additional Measures From Multivariable Model
eReferences

This supplementary material has been provided by the authors to give readers additional information about their work.

eTable 1: Descriptive statistics comparing patients included in study to those excluded due to missing primary outcome i.e. patients transferred from index hospital prior to discharge

	No IV Fluid & Included	No IV Fluid & Excluded	IV Fluid & Included	IV Fluid & Excluded	p-value*	SMD*
n	856	416	1015	453		
Dispatch Priority No.(%)					<0.001	0.32
Omega/Alpha	191 (22)	90 (22)	157 (16)	87 (19)		
Bravo/Charlie	333 (39)	214 (51)	396 (39)	229 (51)		
Delta/Echo	332 (39)	112 (27)	462 (46)	137 (30)		
Community Size No.(%)					0.001	0.19
Metro	665 (78)	313 (75)	850 (84)	372 (82)		
Moderate Metro Influence	47 (5.5)	30 (7.2)	64 (6.3)	25 (5.5)		
Urban	47 (5.5)	17 (4.1)	45 (4.4)	19 (4.2)		
Moderate Urban Influence	6 (0.7)	2 (0.5)	2 (0.2)	1 (0.2)		
Rural Centre	67 (7.8)	39 (9.4)	38 (3.7)	28 (6.2)		
Rural	15 (1.8)	11 (2.6)	15 (1.5)	6 (1.3)		
Rural Remote	9 (1.1)	4 (1.0)	1 (0.1)	2 (0.4)		
Unit Type ALS No.(%)	757 (88)	363 (87)	914 (90)	404 (89)	0.68	0.03
Age median [IQR]	78 [65, 86]	82 [70, 88]	75 [63, 84]	82 [68, 89]	<0.001	0.31
Male Sex No.(%)	394 (46)	185 (45)	522 (51)	236 (52)	0.86	0.01
Weight (kg) mean (SD)	81 (29)	80 (28)	77 (26)	78 (22)	0.71	0.03
SBP ≤ 110 No.(%)	226 (26)	94 (23)	378 (37)	183 (40)	0.45	0.07
Systolic BP mean (SD)	131 (31)	131 (27)	122 (32)	120 (29)	0.13	0.09
Systolic BP Strata					0.26	0.12
<80	28 (3.3)	10 (2.4)	98 (9.7)	38 (8.4)		
80-100	111 (13)	42 (10)	172 (17)	93 (21)		
101-120	713 (83)	361 (87)	735 (72)	320 (71)		
>120	4 (0.5)	3 (0.7)	10 (1.0)	2 (0.4)		
Diastolic BP mean (SD)	75 (21)	73 (20)	70 (21)	68 (19)	0.24	0.07
MAP mean (SD)	93 (21)	92 (19)	86 (23)	84 (22)	0.18	0.08
Respiratory Rate mean (SD)	23 (8.5)	23 (7.9)	26 (10)	25 (8.7)	0.076	0.10
Pulse Oximetry median [IQR]	92 [84, 96]	92 [86, 96]	89 [83, 94]	92 [87, 95]	<0.001	0.28
Heart Rate mean (SD)	98 (25)	95 (25)	102 (27)	103 (27)	0.75	0.02
Temperature mean (SD)	37.0 (1.1)	36.9 (1.1)	37.1 (1.3)	37.2 (1.3)	0.42	0.05
Blood Glucose mean (SD)	8.1 (3.6)	8.2 (3.7)	8.3 (4.3)	8.9 (4.8)	0.057	0.11
GCS median [IQR]	15 [13, 15]	14 [11, 15]	14 [12, 15]	13 [10, 15]	<0.001	0.21
Emergent Transport No.(%)	41 (4.8)	16 (3.8)	121 (12)	76 (17)	0.063	0.14

© 2018 Lane DJ et al. *JAMA Network Open.*

Distance median [IQR]	12 [9, 17]	12 [9, 16]	12 [9, 17]	12 [9, 16]	0.56	0.07
Paramedic Impression Sepsis No.(%)	136 (16)	46 (11)	207 (20)	71 (16)	0.063	0.14
Prehospital Critical Illness Score mean (SD)	2.6 (1.4)	2.7 (1.2)	3.2 (1.4)	3.1 (1.3)	0.22	0.07
IV Rate (%)						
No IV	714 (83)	340 (82)	0 (0.0)	0 (0.0)		
Saline Lock/IV Placed	142 (17)	76 (18)	0 (0.0)	0 (0.0)		
TKVO	0	0	382 (38)	182 (40)		
Bolus	0	0	633 (62)	271 (60)	0.39	0.05
Total IV Volume median [IQR]	0	0	400 [250, 500]	400 [250, 525]	0.76	0.02
Prehospital Time (min) mean (SD)	43 (16)	43 (18)	45 (16)	46 (18)	0.48	0.04
Time to MD Assessment (min) mean (SD)	71 (72)	80 (87)	64 (71)	66 (68)	0.64	0.03

*p-value and SMD (standardized mean difference) comparing IV included to IV excluded groups

IV, intravenous; TKVO, “to-keep-vein-open” rate of infusion; SMD, standardized mean difference; Dispatch Priority, omega/alpha – low priority, delta/echo- high priority; ALS, advanced life support; IQR, interquartile range; SD, standard deviation; BP, blood pressure; MAP, mean arterial pressure; GCS, Glasgow Coma Scale

© 2018 Lane DJ et al. *JAMA Network Open.*

Appendix 1: Propensity-Matched Analysis

Appraisal of the propensity match, and instrumental variable followed recommendations from the Users' Guide to the Medical Literature.[1]

eTable 2: Descriptive characteristics of propensity-matched patient cohort (n=1,489). Patients were matched 1:1 with replacement of controls.

	No IV	IV	SMD
n	474	1,015	
Propensity Score	0.58	0.58	0.00
Age mean	73	71	0.01
Unit Type BLS (%)	53 (11)	101 (10)	0.05
ALS (%)	412 (90)	914 (90)	0.05
Sex Male (%)	238 (51)	522 (51)	0.029
Weight mean	77	77	0.00
Systolic BP mean	122	123	-0.02
Respiratory Rate mean	25	25	-0.04
Pulse Oximetry mean	87	87	0.12
Heart Rate mean	102	102	-0.03
Temperature mean	37.1	37.1	0.10
Blood Glucose mean	8.5	8.4	-0.03
GCS mean	13	13	0.04
Congested on Auscultation No.(%)	67 (14.1)	161 (15.9)	0.048
Crackles on Auscultation No.(%)	73 (15.4)	160 (15.8)	0.01
Wheeze on Auscultation No.(%)	50 (10.5)	111 (10.9)	0.013
Decreased Air Entry on Auscultation No.(%)	108 (22.8)	288 (28.4)	0.128
Clammy Skin No.(%)	39 (8.2)	97 (9.6)	0.047
Pale Skin No.(%)	106 (22.4)	276 (27.2)	0.112
Skin Diaphoretic No.(%)	30 (6.3)	83 (8.2)	0.071
Skin Jaundice No.(%)	7 (1.5)	21 (2.1)	0.045
Abnormal Skin Turgor No.(%)	49 (10.3)	148 (14.6)	0.129
Malaise No.(%)	24 (5.1)	71 (7.0)	0.081
Dyspnea No.(%)	59 (12.4)	109 (10.7)	0.053
Weakness No.(%)	26 (5.5)	54 (5.3)	0.007

IV, intravenous; SMD, standardized mean difference; BP, blood pressure; MAP, mean arterial pressure; GCS, Glasgow Coma Scale

© 2018 Lane DJ et al. *JAMA Network Open.*

eFigure 1: Standardized mean difference of baseline patient characteristics used to generate propensity score.

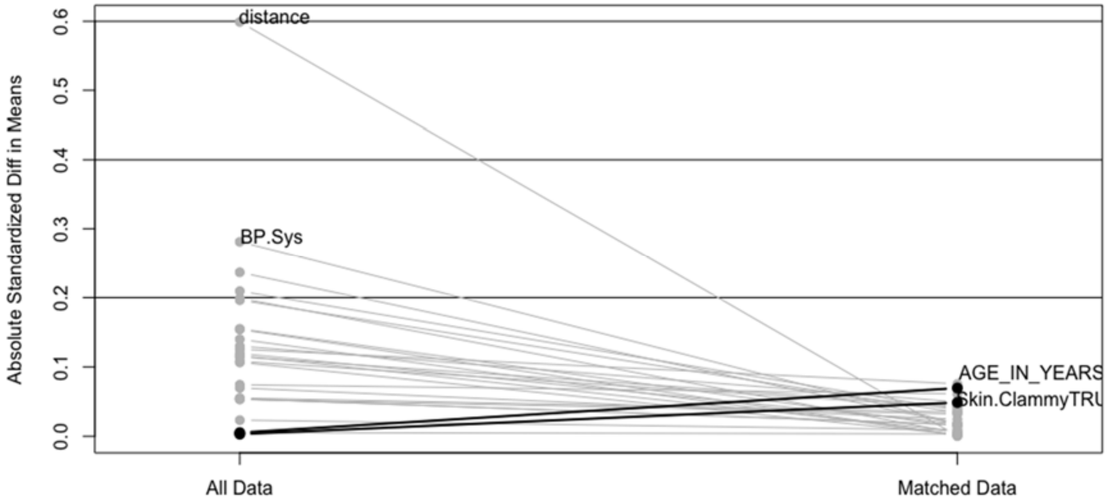

This figure compares the SMD of all variables included in propensity score among patients who received IV treatment to those who did not, before and after they were matched. Overall propensity score referred to as "distance". Grey lines indicate a decrease in SMD, black lines indicate an increase in SMD.

eFigure 2: Changes in odds of mortality for IV fluid treatment at different initial systolic blood pressures, with 95% confidence bands, in propensity matched model

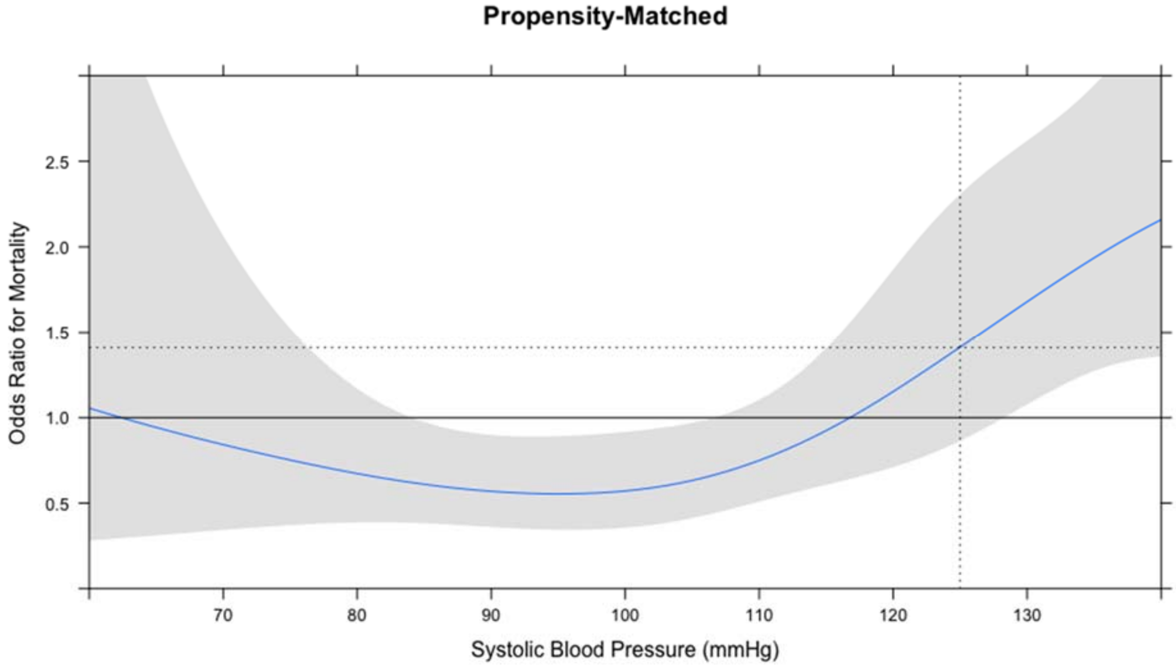

*dotted line indicates cohort median estimate (i.e. adjusted to median systolic blood pressure of 125 mmHg).

© 2018 Lane DJ et al. *JAMA Network Open.*

Appendix 2: Instrumental Variable Analysis

We created an instrumental variable to account for variations in paramedic treatment preferences for administering IV fluid across municipalities, by estimating the probability of receiving an IV and any fluid for each municipality in the study region (n=423 in study cohort). This instrumental variable – the probability of receiving IV fluid treatment per municipality – should be highly associated with the exposure of interest (i.e. intravenous fluid) but not associated with the outcome of interest. Using this instrumental variable, we tested for endogeneity (i.e. residual confounding of the treatment conditional on the exogenous instrumental variable)[2] using the Wu-Hausman test by fitting this instrumental variable as a continuous measure using the two-stage logistic regression approach, with the same baseline patient measures and other exposures.[3] The 'ivreg' function from the AER package for instrumental variable analysis.

Our instrumental variable was associated with IV treatment (OR 1.7 95% CI 1.5-19) and not associated with the outcome (OR 1.0 95% CI 0.97-1.1). However, after adjusting for the baseline patient characteristics we found no evidence of endogeneity for IV treatment (Wu-Hausman p-value = 0.359), therefore the use of an instrumental variable analysis was not necessary. Residual confounding is always a concern in observational studies of treatment effects, but this finding of no evidence of endogeneity suggests the patient characteristics we included in our models accounted for much of this confounding.

eTable 3: Characteristics of patients across instrumental variable tertiles

	1st Tertile	*2nd Tertile*	*3rd Tertile*	*SMD*
n	966	813	91	
Age median [IQR]	77 [65, 86]	75 [63, 85]	74 [64, 84]	0.091
Male Sex No.(%)	478 (49.5)	389 (47.8)	48 (52.7)	0.065
Weight mean (SD)	79 (27)	78 (27)	85 (30)	0.169
Systolic BP ≤110 mmHg (%)	300 (31)	273 (34)	31 (34)	0.058
Systolic BP mean (SD)	128 (33)	125 (31)	124 (28)	0.078
Systolic BP Strata				0.058
<80	67 (6.9)	54 (6.6)	5 (5.5)	
80-100	146 (15)	124 (15)	13 (14)	
101-120	747 (77)	628 (77)	72 (79)	
Diastolic BP mean (SD)	72 (21)	72 (22)	70 (20)	0.082
MAP mean (SD)	90 (23)	89 (22)	89 (21)	0.041
Respiratory Rate mean (SD)	24 (10)	25 (10)	26 (10)	0.121
Pulse Oximetry median [IQR]	90 [84, 95]	90 [83, 95]	88 [79, 95]	0.158
Heart Rate mean (SD)	99 (26)	102 (26)	98 (28)	0.094
Temperature mean (SD)	37 (1.1)	37 (1.2)	37 (1.2)	0.186
Blood Glucose mean (SD)	8.3 (4.3)	8.3 (4.0)	7.6 (2.6)	0.149
GCS median [IQR]	14 [13, 15]	14 [12, 15]	14 [12, 15]	0.065

© 2018 Lane DJ et al. *JAMA Network Open.*

Paramedic Impression Sepsis No.(%)	89 (9.2)	65 (8.0)	8 (8.8)	0.117
Emergent Transport No.(%)	147 (15)	175 (22)	21 (23)	0.157
Prehospital Critical Illness Score mean (SD)	2.9 (1.4)	3.1 (1.4)	3.2 (1.4)	0.145
IV Received No.(%)	449 (47)	494 (61)	72 (79)	0.472
Total IV Volume median [IQR]	0.0 [0.0, 200]	0.0 [0.0, 300]	200 [0.0, 500]	0.286
Prehospital Time (min) mean (SD)	43 (15)	44 (16)	54.1 (22)	0.373
ICU Admission No.(%)	148 (15)	130 (16)	17 (19)	0.06
Length of Stay median [IQR]	7.0 [4.0, 13]	7.0 [3.0, 13]	6.0 [3.5, 11]	0.03
In-Hospital Mortality No.(%)	252 (26)	255 (31)	20 (22)	0.142

IV, intravenous; SMD, standardized mean difference; SD, standard deviation; BP, blood pressure; MAP, mean arterial pressure; GCS, Glasgow Coma Scale; ICU, intensive care unit

This table describes patient characteristics across levels of the instrumental variable, stratified into tertiles to allow comparison. We expect the instrumental variable to be unrelated to other patient characteristics and therefore no differences between instrumental variable stratifications.

eFigure 3: Proportion of patients that received IV fluid across levels of instrumental variable

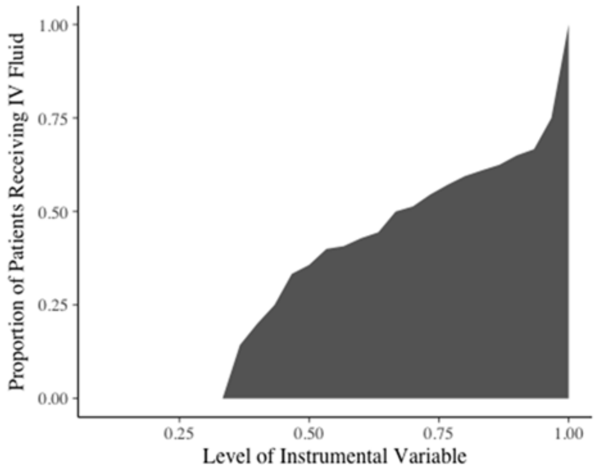

This diagram demonstrates differences in the proportion of patients receiving IV fluid based on the instrumental variable, supporting its use as a surrogate to randomization given the difference across levels. The odds ratio for the association of the instrumental variable with IV fluid (i.e. treatment) was 1.7 95% CI 1.5-1.9. This reflects a moderate association. The odds ratio for the association of the instrumental variable with mortality (i.e. primary outcome) was 1.0 95% CI 0.98-1.1. This reflects no association.

© 2018 Lane DJ et al. *JAMA Network Open.*

eFigure 4: Changes in odds of mortality for IV fluid treatment at different initial systolic blood pressures, with 95% confidence bands, in instrumental variable model

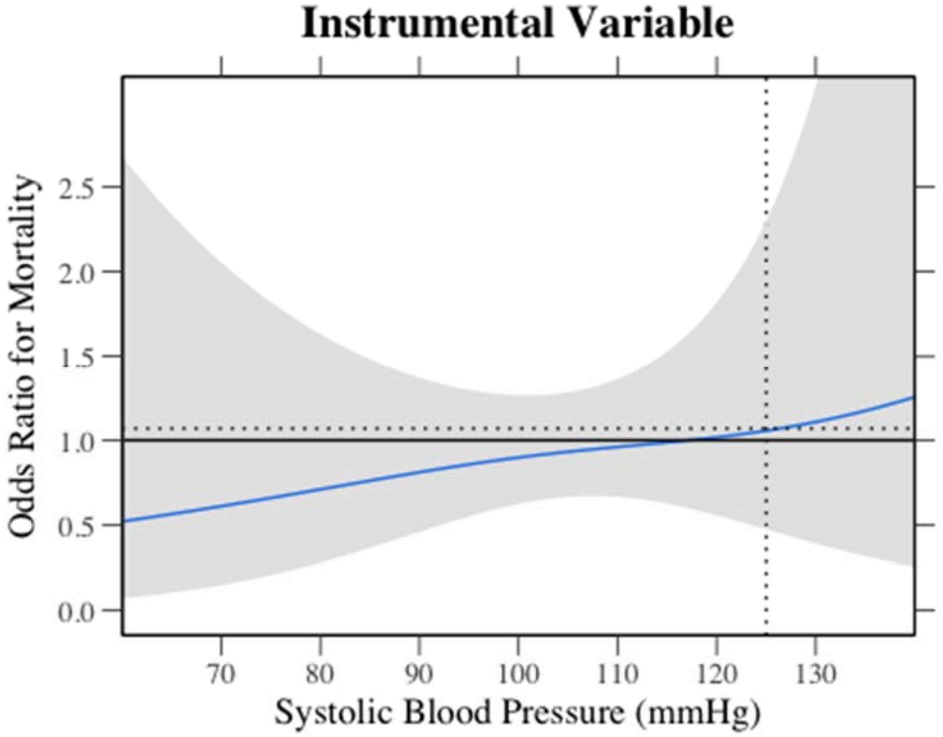

*dotted line indicates cohort median estimate (i.e. adjusted to median systolic blood pressure of 125 mmHg).

eTable 4: Odds ratios (95% CI) for hospital mortality in patients with sepsis treated with IV fluid treatment by paramedics

	Cohort Median		*Hypotensive*		*Hypertensive*	
Adjusted SBP (mmHg)	125		100		140	
Instrumental Variable‡	1.1	(0.48, 2.8)	0.90	(0.63, 1.3)	1.3	(0.26, 6.4)

‡Association of instrument with IV treatment 1.7 (95% CI 1.5-19); Association of instrument with outcome 1.0 (95% CI 0.97-1.1). Weak instruments F test p <0.001. Wu-Hausman test of endogeneity p-value = 0.359.
TKVO, “to-keep-vein-open” rate of infusion

© 2018 Lane DJ et al. *JAMA Network Open.*

eFigure 5: Changes in odds of mortality for IV fluid treatment at different initial systolic blood pressures, with 95% confidence bands, in sensitivity analysis comparing patients who received fluid at a bolus rate to patients who received no IV fluid

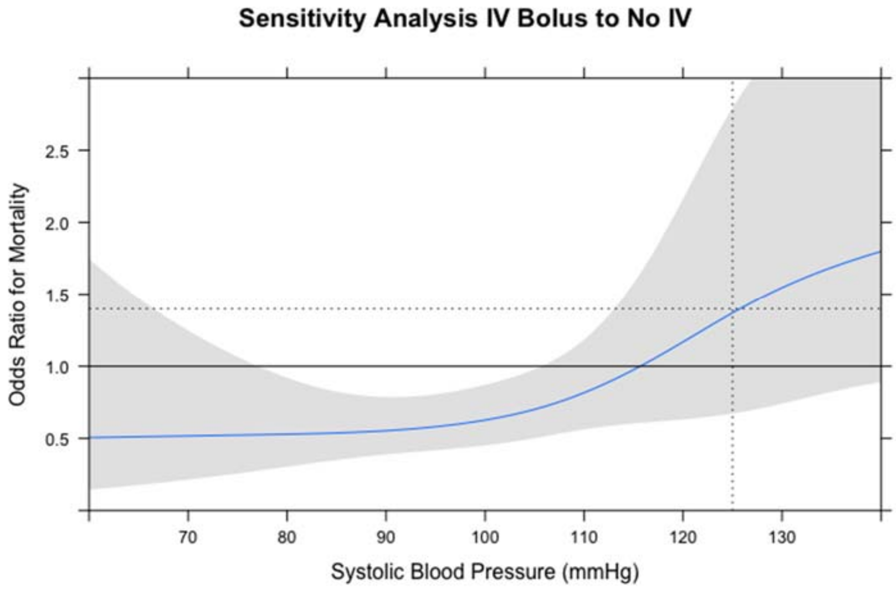

*dotted line indicates cohort median estimate (i.e. adjusted to median systolic blood pressure of 125 mmHg).

© 2018 Lane DJ et al. *JAMA Network Open.*

eFigure 6: Changes in odds of mortality for IV fluid given patients initial systolic blood pressure excluding patients with missing measures

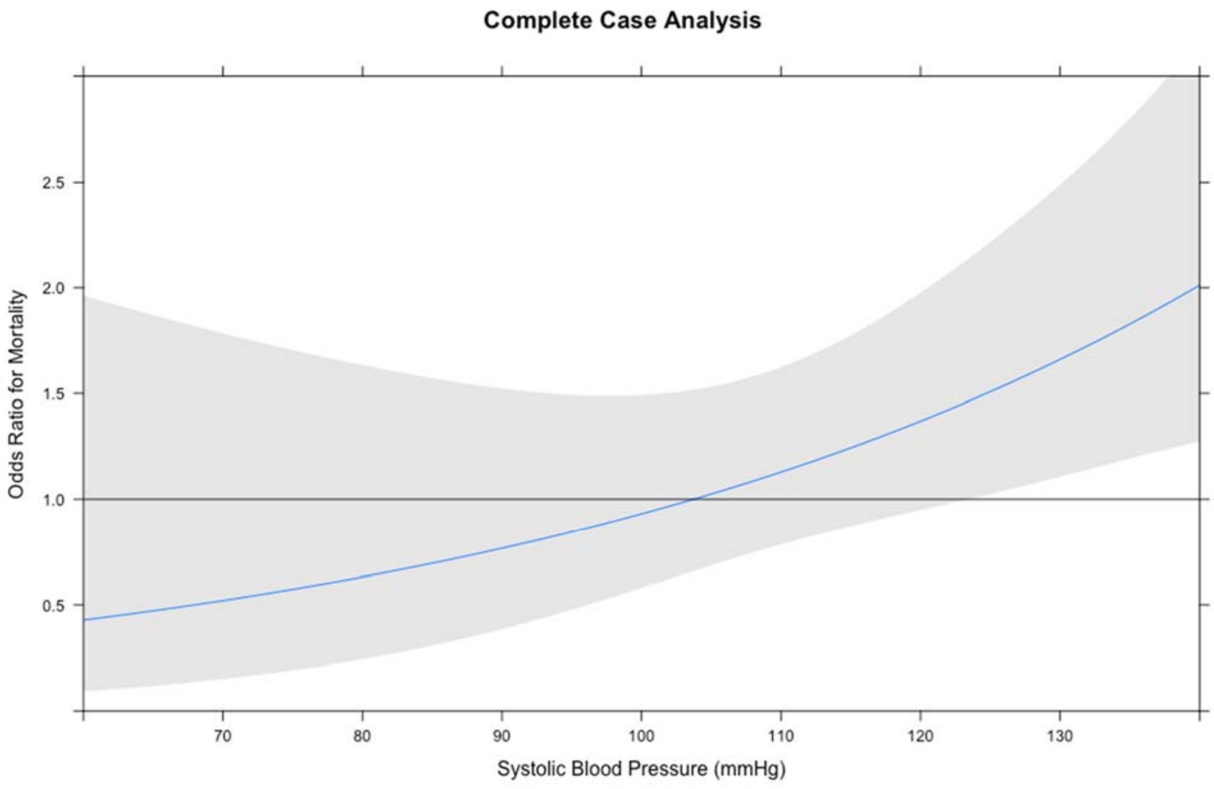

eTable 5: Sensitivity analysis odds ratios (95% CI) for hospital mortality in patients with sepsis treated with IV fluid treatment by paramedics

Excluding Patients with Missing Values (Total n=925)						
Covariate						
Adjusted SBP (mmHg)	125		100		140	
Multivariable Any	1.5	(1.0, 2.2)	0.93	(0.58, 1.5)	2.0	(1.3, 3.2)
*Propensity Matched**	1.5	(0.74, 3.1)	0.92	(0.46, 1.8)	2.7	(1.3, 5.6)
Instrumental Variable	0.99	(0.77, 1.3)	0.82	(0.55, 1.2)	1.2	(0.35, 4.1)
Repeating Analysis as per Seymour et al.						
Multivariable	1.20	(0.96, 1.51)				

*749 patients matched

© 2018 Lane DJ et al. *JAMA Network Open.*

eFigure7: Odds of in-hospital mortality for additional measures from multivariable model

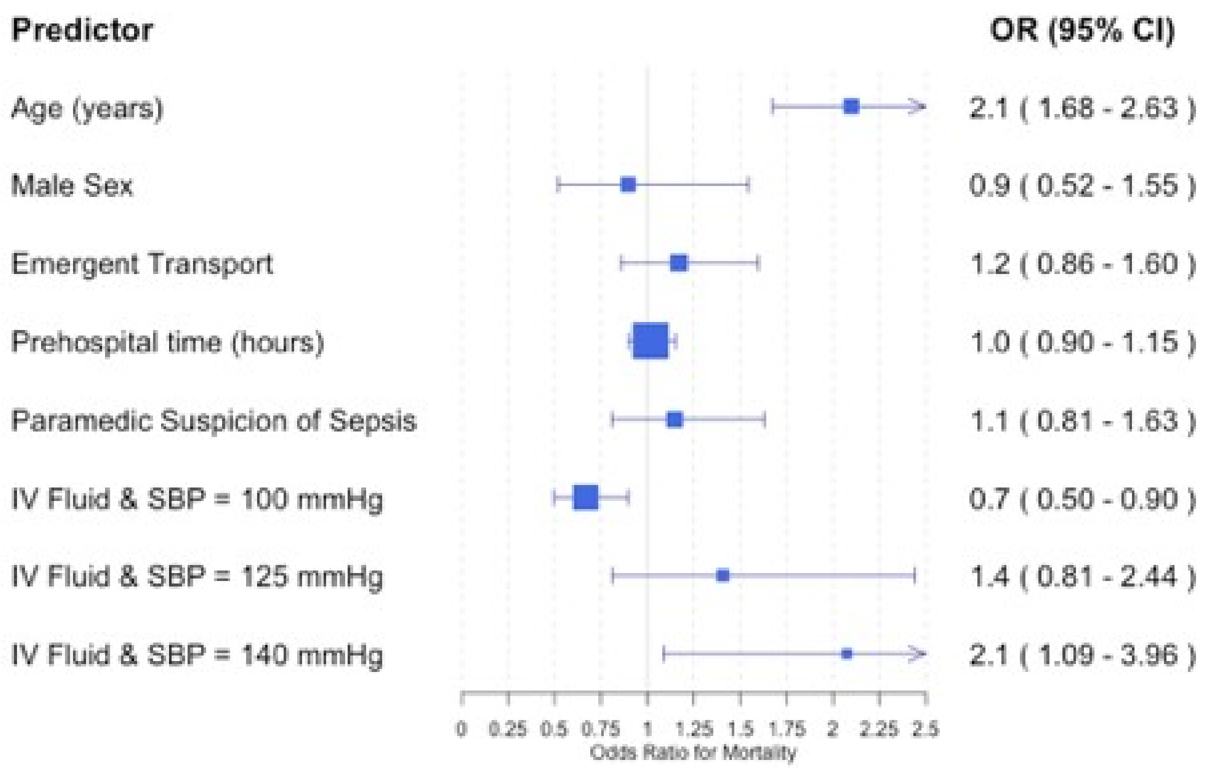

Error bars indicate 95% confidence intervals. Size of square denotes precision of estimate. An x-axis value of 1 denotes no association with mortality. Values to the right of this line indicate increased mortality while values to the left indicate decreased mortality. IV, intravenous; SBP, systolic blood pressure

Appendix 3: R Code

```
sepsis<-subset(EMS, Sepsis==T&AGE_IN_YEARS>=18)

Table1<-CreateTableOne(vars = var, factorVars = catVars, includeNA = T, strata= c("Transfer"), data=sepsis.qSOFA[sepsis.qSOFA$IV.Any==T,], test = T, smd = T)
Table1<-print(Table1, format = "fp",  showAllLevels = F, nonnormal = continuousvars,test = T,smd = T,catDigits=1, contDigits=1)

#Multiple Imputation
col<-c("Card","Month",
"Disp.Priority","URBAN","Unit.Type","AGE_IN_YEARS","SEX","weight","BP.Sys","BP.Dia","MAP.c","RR","SPO2","HR","TEMP","Glucose","GCS.i","BreathSoundsCongested","BreathSoundsCrackles","BreathSoundsWheeze","BreathSoundsDecreasedAir",
```

© 2018 Lane DJ et al. *JAMA Network Open.*

```
"Skin.Clammy","Skin.Pale","Skin.Diaphoretic","Skin.Jaun","Skin.Turgor","Symp.Malaise","Symp.qSOFAusea","S
ymp.Dypsnea","Symp.Diarrhea","Symp.Dizziness","Symp.Fever","Symp.Swelling","Symp.Unresponsive","Symp.
Weakness","Symp.PainGeneral","Paramedic.Impress.Sepsis","Distance","Dist.Centre","Trans.Priority","PHtime.mi
n","PHtime.hour","IV.Any","IV.Rate","IV.TotVol", "Time.to.MD.Assess",
"Time.ED.MD.Assess","AdmitOutcome", "ALL_DAYS" ,"DeathOutcome", "GCS.Strat")

set.seed(1987)
fluid.i<-aregImpute(eval(parse(text= noquote(paste("~",paste(col[-c(1:2,30,37:39,41:49,51)], collapse = "+"))))), data
= sepsis, n.impute = 32, nk=4, x=T,type="pmm")
fluid.i
sepsis<-subset(sepsis, sepsis$DeathNA==F)

Fluid.dd <- datadist(sepsis[c(col)]); options(datadist= "Fluid.dd") #

### Operational Characteristic Models ###
var<-c("AGE_IN_YEARS","SEX","weight",
"RR","SPO2","HR","TEMP","Glucose","GCS.i","BreathSoundsCongested","BreathSoundsCrackles","BreathSound
sWheeze","BreathSoundsDecreasedAir",
"Skin.Clammy","Skin.Pale","Skin.Diaphoretic","Skin.Jaun","Skin.Turgor","Symp.Malaise","Symp.Dypsnea","Sym
p.Fever","Symp.Unresponsive","Symp.Weakness")
vars<-paste(var, collapse="+")
#What is the average volume difference between patients recieving IV fluid, after controlling for covariates expected
to contribute to variation?

totvol<-areg.boot(eval(parse(text= noquote(paste0("IV.TotVol~",vars,
paste0("+BP.Sys+Trans.Priority+Paramedic.Impress.Sepsis+URBAN+Unit.Type"))))), method="avas", data =
sepsis, B=200)

summary(totvol, values =list(Paramedic.Impress.Sepsis=c(0, 1), Trans.Priority=c(0, 1), BP.Sys=c(125,100,140)))

### Question 2: Does treatment contribute to longer prehospital times?
time<-areg.boot(eval(parse(text= noquote(paste0("PHtime.min~",vars,
paste0("+IV.Rate*BP.Sys+Trans.Priority+Paramedic.Impress.Sepsis+URBAN+Unit.Type+Month+Time.Call"))))),
method="avas", data = sepsis, B=200)
time
summary(time,values =list(Paramedic.Impress.Sepsis=c(0, 1), IV.Any=c(0, 1),BP.Sys=c(125,100,140)))
```

© 2018 Lane DJ et al. *JAMA Network Open.*

```
#Time to MD
time<-areg.boot(eval(parse(text= noquote(paste0("Time.ED.MD.Assess~",vars,
paste0("+IV.Any*BP.Sys+Trans.Priority+Paramedic.Impress.Sepsis+URBAN+Unit.Type+Month+Time.Call+PHti
me.min"))))),method="avas", data = sepsis, B=200)
summary(time,values =list(Paramedic.Impress.Sepsis=c(0, 1), IV.Any=c(0, 1),BP.Sys=c(125,100,140)))

### Question 3: Does treatment reduce hospital mortality?

###Primary Analysis ###
#Repeating Seymour Study
mortality<-
fit.mult.impute(DeathOutcome~IV.Any+AGE_IN_YEARS+SEX+HR+RR+GCS.i+SPO2+BP.Sys+Trans.Priority+
PHtime.hour+Disp.Priority, data=sepsis, lrm, fluid.i)

### Multivariable Model###
var<-c("Trans.Priority","Paramedic.Impress.Sepsis","rcs(AGE_IN_YEARS,3)","Unit.Type","SEX","GCS.i",
"rcs(RR,3)","rcs(SPO2,3)","rcs(HR,3)","TEMP","rcs(Glucose,3)","BreathSoundsCongested","BreathSoundsCrackle
s","BreathSoundsWheeze","BreathSoundsDecreasedAir",
"Skin.Clammy","Skin.Pale","Skin.Diaphoretic","Skin.Jaun","Skin.Turgor","Symp.Malaise","Symp.Dypsnea","Sym
p.Weakness", "URBAN", "weight")
vars<-paste(var, collapse="+")

mortality.mv<-fit.mult.impute(eval(parse(text=
noquote(paste0("DeathOutcome~",paste("IV.Any*rcs(BP.Sys,5)+PHtime.hour+"),vars )))), data=sepsis, lrm, fluid.i)
mortality.mv<-update(mortality.mv, x=T, y=T)
mortality.clus.mv<-robcov(mortality.mv, cluster = sepsis$Institution)
summary(mortality.clus,BP.Sys=125)

k<-contrast(mortality.clus.mv, list(BP.Sys=60:140, IV.Any=T),list(BP.Sys=60:140, IV.Any=F))
xYplot(Cbind(exp(Contrast),exp(Lower),exp(Upper))~BP.Sys, data=k,xlab='Systolic Blood Pressure (mmHg)',
ylab='Odds Ratio for Mortality', main="Multivariable" ,abline=list(list(h=1), list(h=1.4, lty=3), list(v=125, lty=3))
,xlim = c(60,140), ylim=c(0,3),method="filled bands",col.fill="gray90",type="l", scales=list(tick.number=10))

#IV Rate
k<-contrast(mortality.clus, list(BP.Sys=60:140, IV.Rate="Bolus"),list(BP.Sys=60:140, IV.Rate="No IV"))
xYplot(Cbind(exp(Contrast),exp(Lower),exp(Upper))~BP.Sys, data=k,xlab='Systolic Blood Pressure (mmHg)',
ylab='Odds Ratio for IV Treatment and Mortality', main="Propensity-Matched" ,abline=list(list(h=1), list(h=0.98,
```

© 2018 Lane DJ et al. *JAMA Network Open.*

```
lty=3), list(v=125, lty=3)) ,xlim = c(60,140), ylim=c(0,3),method="filled bands",col.fill="gray90",type="l",
scales=list(tick.number=10))

#Forest Plot of select estimates - eFigure 1
overall<-summary(mortality.clus, IV.Any=T)
hypo<-summary(mortality.clus, BP.Sys=100)
hyper<-summary(mortality.clus, BP.Sys=140)
names<-c("Age (years)","Male Sex","Emergent Transport","Prehospital time (hours)","Paramedic Suspicion of
Sepsis","IV Fluid & SBP = 100 mmHg", "IV Fluid & SBP = 125 mmHg", "IV Fluid & SBP = 140 mmHg")

OR.Estimates <- structure(list(
  OR  = c(overall[12,4],overall[54,4],overall[8,4],overall[6,4],overall[10,4],hypo[2,4],overall[2,4], hyper[2,4]),
  OR.lower = c(overall[12,6],overall[54,6],overall[8,6],overall[6,6],overall[10,6],hypo[2,6],overall[2,6], hyper[2,6]),
  OR.upper = c(overall[12,7],overall[54,7],overall[8,7],overall[6,7],overall[10,7],hypo[2,7],overall[2,7],
hyper[2,7])),
  .qSOFAmes = c("Odds Ratio", "lower", "upper"),
  row.qSOFAmes = paste(names),
  class = "data.frame")

library(forestplot)
forestplot(OR.Estimates,
        is.summary=c(rep(FALSE,length(names))),
        xlab = "Odds Ratio for Mortality", zero = 1,
        grid=T, clip=c(0,2.5), xticks = c(0, 0.25, 0.5, 0.75, 1, 1.25, 1.5, 1.75, 2, 2.25, 2.5),
        txt_gp = fpTxtGp(cex=1.5, ticks=gpar(cex=1.0), xlab = gpar(cex=1.0)),
        col=fpColors(box="royalblue",line="darkblue", summary="royalblue", hrz_lines = "#444444"),
        vertices = TRUE)

#Propensity Matched
library(MatchIt)
var<-
c("AGE_IN_YEARS","Unit.Type","SEX","BP.Sys","RR","SPO2","HR","TEMP","GCS.i","BreathSoundsCongeste
d","BreathSoundsCrackles","BreathSoundsWheeze","BreathSoundsDecreasedAir",
"Skin.Clammy","Skin.Pale","Skin.Diaphoretic","Skin.Jaun","Skin.Turgor","Symp.Malaise","Symp.Dypsnea","Sym
p.Weakness","weight", "URBAN")
vars<-paste(var, collapse = "+")
```

© 2018 Lane DJ et al. *JAMA Network Open.*

```
others<-c("IV.Any","IV.Rate","PHtime.hour","Paramedic.Impress.Sepsis","Trans.Priority","DeathOutcome",
"Institution",
"URBAN","Glucose","Disp.Priority","BP.Dia","MAP.c","Symp.qSOFAusea","Symp.Dizziness","Symp.Fever","Sy
mp.Swelling","Symp.Unresponsive","Symp.PainGeneral")

matched<-matchit(eval(parse(text= noquote(paste0("IV.Any~",vars)))), data =sepsis[c(var, others)],
method="nearest",ratio=1,replace=T)
matched

match.char<-summary(matched,standardize=T)
sepsis.propensity<-match.data(matched, group="all")
mortality.pm<-
lrm(DeathOutcome~IV.Any*rcs(BP.Sys,5)+PHtime.hour+Paramedic.Impress.Sepsis+Trans.Priority+URBAN,
data=sepsis.propensity, weights = weights, x=T, y=T)
mortality.pm<-
fit.mult.impute(DeathOutcome~IV.Any*rcs(BP.Sys,5)+PHtime.hour+Paramedic.Impress.Sepsis+Trans.Priority+UR
BAN,lrm, fluid.i, data=sepsis.propensity)
mortality.pm<-update(mortality.pm, x=T, y=T)
mortality.clus.pm<-robcov(mortality.pm, cluster = sepsis.propensity$Institution)
summary(mortality.clus,  BP.Sys=140)

k<-contrast(mortality.pm, list(BP.Sys=60:140, IV.Any=T),list(BP.Sys=60:140, IV.Any=F))
xYplot(Cbind(exp(Contrast),exp(Lower),exp(Upper))~BP.Sys, data=k,xlab='Systolic Blood Pressure (mmHg)',
ylab='Odds Ratio for Mortality', main="Propensity-Matched" ,abline=list(list(h=1), list(h=1.41, lty=3), list(v=125,
lty=3)) ,xlim = c(60,140), ylim=c(0,3),method="filled bands",col.fill="gray90",type="l",
scales=list(tick.number=10))

#Instrumental variable
var<-c("Trans.Priority","Paramedic.Impress.Sepsis","rcs(AGE_IN_YEARS,3)","Unit.Type","SEX","rcs(weight,3)",
"rcs(RR,3)","SPO2","rcs(HR,3)","TEMP","rcs(Glucose,3)","GCS.i","BreathSoundsCongested","BreathSoundsCrac
kles","BreathSoundsWheeze","BreathSoundsDecreasedAir",
"Skin.Clammy","Skin.Pale","Skin.Diaphoretic","Skin.Jaun","Skin.Turgor","Symp.Malaise","Symp.Dypsnea","Sym
p.Weakness", "URBAN")
vars<-paste(var, collapse="+")
instrumental<-fit.mult.impute(IV.Any~Municipality, data=sepsis, lrm, fluid.i)
sepsis$IV.instrumental<-predict(instrumental, type = "fitted")
```

© 2018 Lane DJ et al. *JAMA Network Open.*

```
prop<-data.frame(CrossTable(sepsis$IV.instrumental,sepsis$IV.Any)$prop.row)
prop$x<-as.numeric(prop$x)
library(scales)
prop$Instrumental<-rescale(as.numeric(prop$x), to=c(0,1))
ggplot(prop[35:68,], aes(Instrumental))+
  geom_area(aes(y=Freq),fill = "grey40")+
  xlab("Level of Instrumental Variable")+
  ylab("Proportion of Patients Receiving IV Fluid")

library(AER)
library(ivpack)

mortality<-ivreg(eval(parse(text=
noquote(paste0("DeathOutcome~",paste("IV.Any*rcs(BP.Sys,3)+PHtime.hour+Paramedic.Impress.Sepsis+"),vars,p
aste("|.-IV.Any+IV.instrumental*BP.Sys"))))), data=sepsis, x=T)
anderson.rubin.ci(mortality)
summary(mortality, vcov = sandwich, diagnostics = TRUE)

#eFigure6
IV.OR<-matrix(nrow = 81, ncol=4)
for(i in 1:81){
  bp<-i+59
  sepsis$BP<-sepsis$BP.Sys-bp
  model<-ivreg(eval(parse(text=
noquote(paste0("DeathOutcome~",paste("IV.Any*rcs(BP,3)+PHtime.hour+Paramedic.Impress.Sepsis+"),vars,paste(
"|.-IV.Any+IV.instrumental*BP"))))), data=sepsis, x=T)
  CI<-anderson.rubin.ci(model)
  low<-exp(as.numeric(substr(CI, start = 2, stop = regexpr(",", CI)[1]-1)))
  up<-exp(as.numeric(substr(CI, start = regexpr(",", CI)[1]+1, stop = nchar(CI)-1)))
  IV.OR[i,]<-cbind(bp, exp(coef(model)[2]), low, up)
}
colnames(IV.OR)<-c("BP", "OR", "lower", "upper")
IV.OR<-as.data.frame(IV.OR)
scaleFUN <- function(x) sprintf("%.1f", x)
ggplot()+
  geom_line(data=IV.OR, aes(x=BP, y=OR), color="dodgerblue")+
  geom_hline(yintercept = 1)+
```

© 2018 Lane DJ et al. *JAMA Network Open.*

```
  geom_hline(yintercept = 1.07, lty=3)+
  geom_vline(xintercept = 125, lty=3)+
  geom_ribbon(data = IV.OR, aes(x=BP, ymin=lower, ymax=upper), alpha=0.15, col="gray90")+
  labs(title="Instrumental Variable",
       y="Odds Ratio for Mortality",
       x="Systolic Blood Pressure (mmHg)")+
  scale_x_continuous(expand = c(0,0), breaks=seq(70, 130, by=10),labels =seq(70, 130, by=10),
                     sec.axis = dup_axis(labels = NULL, name = NULL))+
  scale_y_continuous(labels=scaleFUN, breaks=seq(0,2.5, by=0.5),sec.axis = dup_axis(labels = NULL, name =
NULL))+
  theme(text = element_text(size=13), axis.ticks.length= unit(0.25, "cm"),
        panel.border = element_rect(colour = "black", fill=NA, size=0.5),
        plot.title = element_text(face="bold", hjust=0.5, size=16))+
  coord_cartesian(ylim = c(0, 3), xlim = c(60,140))
```

© 2018 Lane DJ et al. *JAMA Network Open.*

eReferences

1. Agoritsas T, Merglen A, Shah ND, O'Donnell M, Guyatt GH. Adjusted Analyses in Studies Addressing Therapy and Harm. *JAMA*. 2017;317(7):748–12. doi:10.1001/jama.2016.20029.

2. Bound J, Jaeger DA, Baker RM. Problems with Instrumental Variables Estimation when the Correlation between the Instruments and the Endogenous Explanatory Variable is Weak. *Journal of the American Statistical Association*. 2012;90(430):443-450. doi:10.1080/01621459.1995.10476536.

3. Klugel O, Jamal Uddin M, de Boer A, Belitser SV, Groenwold RH, Roes KC. Instrumental Variable Analysis in Epidemiologic Studies: An Overview of the Estimation Methods. *Pharmaceutica Analytica Acta*. 2015;06(04). doi:10.4172/2153-2435.1000353.

© 2018 Lane DJ et al. *JAMA Network Open.*
